# Does late water deficit induce root growth or senescence in wheat?

**DOI:** 10.3389/fpls.2024.1351436

**Published:** 2024-06-07

**Authors:** Kanwal Shazadi, John T. Christopher, Karine Chenu

**Affiliations:** The University of Queensland, Queensland Alliance for Agriculture and Food Innovation (QAAFI), Gatton, QLD, Australia

**Keywords:** genotypic variation, root architecture, root development, root growth, root senescence, plant adaptation, drought, wheat

## Abstract

In crops like wheat, terminal drought is one of the principal stress factors limiting productivity in rain-fed systems. However, little is known about root development after heading, when water uptake can be critical to wheat crops. The impact of water-stress on root growth was investigated in two wheat cultivars, Scout and Mace, under well-watered and post-anthesis water stress in three experiments. Plants were grown outside in 1.5-m long pots at a density similar to local recommended farming practice. Differences in root development were observed between genotypes, especially for water stress conditions under which Scout developed and maintained a larger root system than Mace. While under well-watered conditions both genotypes had shallow roots that appeared to senesce after heading, a moderate water stress stimulated shallow-root growth in Scout but accelerated senescence in Mace. For deep roots, post-heading biomass growth was observed for both genotypes in well-watered conditions, while under moderate water stress, only Scout maintained net growth as Mace deep roots senesced. Water stress of severe intensity affected both genotypes similarly, with root senescence at all depths. Senescence was also observed above ground. Under well-watered conditions, Scout retained leaf greenness (i.e. stay-green phenotype) for slightly longer than Mace. The difference between genotypes accentuated under moderate water stress, with rapid post-anthesis leaf senescence in Mace while Scout leaf greenness was affected little if at all by the stress. As an overall result, grain biomass per plant (‘yield’) was similar in the two genotypes under well-watered conditions, but more affected by a moderate stress in Mace than Scout. The findings from this study will assist improvement in modelling root systems of crop models, development of relevant phenotyping methods and selection of cultivars with better adaptation to drought.

## 1 Introduction

Wheat cultivation in rain-fed agricultural systems is commonly challenged by water stress, especially during late crop development (Richter and Semenov, 2005; Farooq et al., 2009; Chenu et al., 2013; Collins and Chenu, 2021). Water stress affects many physiological traits both above and below ground with effects that depend on the timing, the intensity and the duration of the stress (Blum and Sullivan, 1997; Frensch, 1997; Munns, 2002; Collins et al., 2021; Mathew and Shimelis, 2022; Vadez et al., 2024).

One avenue to increase drought tolerance is to breed for varieties with increased capacity to extract soil water. While improving root architecture can assist crops access moist soil layers, the dynamic of root development can also be important to allow water uptake at critical stages (e.g. Veyradier et al., 2013). Beneficial root traits vary depending on the target population of environments. It has been suggested that in environments with shallow soils and frequent low-intensity rainfall, developing a dense root system in shallow soil layers may be advantageous to crops (Gregory et al., 1978; López-Castañeda and Richards, 1994). By contrast, in environments where crops rely heavily on water stored deep in the soil such as in north-eastern Australia (Chenu et al., 2011 and Chenu et al., 2013), a deep root system can benefit crops under late water stress.

Access to water late in the season is particularly important for grain filling. At that stage, a relatively small amount of subsoil water can translate to a major yield gain when crops are water stressed (Kirkegaard et al., 2007; Veyradier et al., 2013). In wheat, crop simulations have shown that an additional mm of water used after anthesis can lead to an extra 60 kg ha^-1^ of grain yield (MansChadi et al., 2006 and MansChadi et al., 2010), and similar results have since been observed in field experiments (Kirkegaard et al., 2007). Candidate traits to increase water extraction at depth include higher root-length density at depth and more uniform root distribution at depth (MansChadi et al., 2006; Asseng and Turner, 2007; Lopes and Reynolds, 2010; Lilley and Kirkegaard, 2011; Ober et al., 2014). Maintaining water uptake under terminal drought typically helps to keep the canopy functional (Christopher et al., 2008) and increase yield (Dodd et al., 2011). Christopher et al. (2008) found that genotypes with contrasting root architecture at depth had different ability to retain green leaf area during the grain filling period (i.e. stay-green phenotype). Multiple studies have shown that post-anthesis water stress is associated with decreased photosynthetic capacity due to early leaf senescence (e.g. Yang et al., 2001), and that stay-green genotypes tend to yield more than others under terminal drought (Christopher et al., 2014 and Kumar et al., 2010; Christopher et al., 2016). Overall however, although higher root biomass and root distribution deeper in the profile has commonly been associated with better adaptation to water stress, little is known about the dynamics and genotypic variability of late root development both in well-watered and water-stress conditions (Palta et al., 2011).

The aims of this study were (i) to characterize wheat post-heading root growth over time in well-watered conditions, (ii) to determine whether post-heading root growth or root senescence occur in response to drought, and (iii) to determine whether any such responses vary between genotypes. Three experiments were conducted with two Australian wheat cultivars with contrasting seedling root systems. The root systems of these cultivars were examined at key stages from heading to maturity in well-watered conditions and in a range of post-anthesis water-stress treatments. A system of 1.5-m polyvinyl chloride (PVC) pots was used to investigate phenotypic differences in both above- and below-ground responses to water stress up to physiological maturity.

## 2 Materials and methods

### 2.1 Growing conditions and experimental design

Three experiments were conducted with two wheat cultivars (Mace and Scout) grown in 1.5-m long polyvinyl chloride (PVC) pots of 90 mm diameter under different soil water conditions. Pots were placed in an outdoor open area in Toowoomba, Queensland, Australia (27.5598°S, 151.9507°E, 691 meters a.s.l.) and arranged to have a plant density similar to local grower’s field (100 plant m^-2^). Furthermore, additional pots were positioned all around the experiment to avoid border effects (Rebetzke et al., 2014).

Mace and Scout are widely cultivated in western and southern cropping regions of Australia, respectively and differ in root architecture at the seedling stage, as Mace has wide root angle while Scout has narrow root angle (Richard et al., 2018). Three seeds of each genotype were placed in each pot at a depth of 2 cm. Sowing occurred on 4 August 2020 in the Experiment 1 (E1), 24 August 2021 in Experiment 2 (E2) and 4 July 2019 in Experiment 3 (E3). Following emergence, plants were thinned to one seedling per pot.

Seeds were sown in a packed soil consisting of a 50:50 mixture of a red alluvial soil from Redlands (-27.53°S, 153.25°E, ~20 m a.s.l.) and black vertosol soil from Kingsthorpe (27.51°S, 152.10°E, ~480 m a.s.l.). To ensure non-limiting nutrient supply, 2 gm L^-1^ of Osmocote fertilizer containing trace elements (N 15.3% P 1.96%, K 12.6%) was added to the soil mix. The soil was watered to pot soil capacity at sowing.

Experimental treatments were denominated by the experiment number (E1, E2, E3) followed by “WW” for well-watered, “MDE” for moderate drought early in grain filling, “MDM” for moderate drought mid-grain filling or “SD” for severe drought with water being withheld from head emergence to maturity (**Table 1**). A cover was placed above the plants before any rainfall events and removed shortly after. Growth stages of individual replicate plants were monitored, and watering withheld between the required developmental periods (**Table 1**).

**Table 1:** Characteristics for experiments (Exp.), treatments (Tmt) and sampling times, including the Zadoks developmental stages between which irrigation was withheld, number of days from sowing to anthesis (Anthesis), and from sowing to maturity (Maturity), green leaf area of the plant at heading (Z50) and grain yield per plant at maturity (Z92) for Mace and Scout.

Exp.	Sowing	Tmt*	Water withholding period**	Genotype	Sampling Time***	Anthesis (Z65) (days)	Maturity (Z92) (days)	Leaf area (Z50) (cm^2^ plt^-1^)	Yield (Z92) (g plt^-1^)
E1	4/08/2020	E1- WW	none	Mace	Z50, Z65, Z92	77 ± 0.9^ns^	129 ± 0.8^ns^	684 ± 55^b^	16 ± 1.5^ns^
				Scout		83 ± 0.8^ns^	131 ± 0.9^ns^	834 ± 78^a^	14 ± 1.5^ns^
	4/08/2020	E1-MDE	Z60–71	Mace	Z50, Z65, Z92	75 ± 0.9^b^	117 ± 0.8^b^	−	7 ± 1.6^b^
				Scout		80 ± 0.8^a^	133 ± 0.9^a^		16 ± 1.8^a^
	4/08/2020	E1-MDM	Z71–81	Mace	Z50, Z65, Z92	78 ± 0.9^b^	117 ± 0.8^b^	−	8 ± 1.8^b^
				Scout		85 ± 0.8^a^	131 ± 0.9^a^		12 ± 1.5^a^
E2	24/08/2021	E2-WW	none	Mace	Z75, Z92	68 ± 1.9^ns^	106 ± 1.9^ns^	−	12 ± 0.4^ns^
				Scout		74 ± 2.2^ns^	110 ± 2.2^ns^		13 ± 0.5^ns^
	24/08/2021	E2-MDE	Z60–71	Mace	Z75, Z92	65 ± 1.9^b^	96 ± 1.9^b^	−	8 ± 0.4^ns^
				Scout		75 ± 2.2^a^	119 ± 1.9^a^		14 ± 0.4^ns^
E3	4/07/2019	E3-SD	Z50–92	Mace	Z50, Z92	79 ± 0.8^ns^	115 ± 0.78^ns^	395 ± 48^ns^	2.5 ± 0.19^ns^
				Scout		86 ± 0.8^ns^	120 ± 0.78^ns^	573 ± 48^ns^	3 ± 0.19^ns^

Average and standard errors were presented for days to anthesis and maturity as well leaf area and yield (n=8). Means followed by different superscript letters are significantly different between genotypes within each treatment (P<0.05), “ns” indicates that the difference between genotypes is not significant (P>0.05). Each experiment was analyzed separately.

*Experimental treatments (Tmt) were denominated by the experiment number followed by “WW” for well-watered, “MDE” for moderate drought early in grain filling, “MDM” for moderate drought mid-grain filling or “SD” for severe drought during grain filling.

**Period of withholding watering are indicated by the number of the Zadoks growth stage from when watering was discontinued followed by the stage where watering was recommenced.

***Harvest of plants occurred at heading (Z50), anthesis (Z65), mid grain filling (Z75) and/or physiological maturity (Z92).

In the first experiment (E1), three irrigation treatments were applied; (i) well-watered conditions during the whole crop cycle (E1-WW); (ii) well-watered conditions followed by a water deficit applied by withholding irrigation between early anthesis (Zadoks decimal growth stage 61; Z61) (Zadoks et al., 1974) and early grain filling (Z71) (E1-MDE), i.e. irrigation stopped 9.9 ± 0.3 days, and (iii) a water deficit imposed by withholding irrigation from early grain filling (Z71) to mid-grain filling (Z81) (E1-MDM), i.e. irrigation stopped for 10.0 ± 0.0 days. In this experiment, plants were harvested at heading (Z50), anthesis (Z65) and maturity (Z92). In a second experiment (E2), two irrigation treatments were applied; (i) well-watered conditions during the whole crop cycle (E2-WW); (ii) well-watered conditions followed by a water deficit applied by withholding irrigation between early anthesis (Z61) and early grain filling (Z71) (E2-MDE), i.e. irrigation stopped for 9.9 ± 0.3 days. In this experiment, plants were harvested at mid-grain filling (Z75) and maturity (Z92). In a third experiment (E3), water was withheld for the whole period from head emergence (Z50) to maturity (Z92) (E3-SD), i.e. irrigation stopped for 53.3 ± 2.1 days. In this experiment, plants were harvested at heading (Z50) and maturity (Z92). For all experiments, pots were watered weekly up to saturation, and let to drained. For each individual pot, watering was also performed up to saturation one day prior to the target growth stage, i.e. the stress was imposed at a date that varied between cultivar, treatments, and repetition). After the water-stress period, plants were rewatered up to saturation when they reached the target stage (**Table 1**). They were watered weekly up to saturation thereafter.

For each experiment, a randomized complete block design was used with eight replicates per cultivar for each treatment and each harvesting time (i.e. heading, anthesis, mid grain filling and/or physiological maturity), a replication being a single plant in a pot.

Environmental characterizations of the three experiments are presented in **Table 2**.

**Table 2:** Environmental conditions in the three experiments (Exp.), including average temperature (Avg. Temp.), average daily maximum temperature (Tmax), average daily minimum temperature (Tmin), average daily evaporation (Avg. Evap.), average daily radiation (Avg. Radn) and cumulated radiation (Cum. Radn) from sowing to maturity of the last maturing plant.

Exp.	Avg. Temp. (°C)	Tmax (°C)	Tmin (°C)	Avg. Evap. (mm)	Avg. Radn (W m^-^²)	Cum. Radn (W m^-^²)
E1	19	25	13	5.1	19	20.1
E2	18.5	24	13.2	4.5	18.5	19.4
E3	17	22.5	10	4.6	17	18.4

### 2.2 Plant measurements

Phenological development was monitored regularly by recording the growth stage using the Zadoks growth scale throughout the experiments (Zadoks et al., 1974). The greenness of the center of the flag leaf of the main stem was measured for each plant (i.e. eight replicates for each cultivar and treatment) at Z50, Z65, Z75 and Z81 using a Minolta SPAD 502 meter (Konica Minolta, Tokyo).

For each harvest, the shoots were excised at the crown. To maintain the root distribution, roots were washed and recovered on a nail board, with nails spaced every 20 mm. Only very few fine roots were lost in the process. Root sections were excised at 10-cm intervals for measurement of dry root biomass. The root biomass was measured following drying for 72h at 70˚C. Total root biomass was calculated as the sum of dry weights for all 10 cm samples for each core. Average root diameter and root length were measured for a subset of soil layers (0–10 cm, 10–20 cm, and alternate 10-cm depth intervals there after (i.e., 30–40, 50–60, 70–80, 90–100, 110–120, and 130–140 cm) using WinRhizo Regular 2019. For each 10-cm depth, average root length density was calculated by dividing the total root length in a segment by the corresponding soil volume. To measure the differences in the partitioning of biomass between shallow, mid and deep roots, root fractions from 0–50 cm were summed to represent shallow roots, 50–100 cm to represent mid roots, and 100–150 cm to represent deep roots. Similarly, average root diameter and root length density were estimated for shallow, mid and deep roots by averaging values from studied depths (0–10, 10–20 and 30–40 cm for shallow roots; 50–60, 70–80 cm for mid roots; and 90–100, 110–120 and 130–140 cm for deep roots). A small proportion of adventitious or nodal roots were present in the shallowest layer (0–10 cm) but not in other layers. Adventitious roots have a greater root diameter than the seminal roots (Christopher, 2024). However, the few adventitious roots were not separated and likely had only a small influence on the mean root diameter values calculated for the upper layers (0 to 50 cm).

The root: shoot ratio was computed by dividing total dry root biomass by the total dry shoot biomass. The total plant biomass was computed by combining the total dry shoot and dry root biomass.

For harvests at heading (Z50) and anthesis (Z65), leaf blades were separated for measurement of green leaf area using a leaf area meter (LI-3000, Li-COR Bioscience, Lincoln, NE, USA).

After threshing spikes by hand, yield was recorded at maturity as the total grain biomass (air dried) per plant.

### 2.3 Statistical analyses

Within each experiment, an analysis of variance (Figueroa-Bustos et al., 2020) was performed between genotypes, treatments and stages for total plant biomass, dry shoot biomass, total dry root biomass, average root length density, average root diameter, dry root biomass at depths, average root length density at depths and average root diameter at depths using the R platform (v3.2.5; Team, R.C, 2019). A Student–Newman–Keuls (SNK) test was used to compare means for genotypes and treatments, with a significance level of 0.05.

## 3 Results

### 3.1 Water stress reduced the duration of the plant growth cycle in Mace but not in Scout

Under well-watered conditions, the plant growth duration from sowing to anthesis and to maturity was slightly shorter for Mace than for Scout, but differences were not significant (**Table 1**).

Application of a post-anthesis moderate water stress shortened the duration from sowing to maturity in Mace but not Scout. While under well-watered conditions the difference in time to maturity between genotypes was relatively small at 2 or 4 days in Experiments 1 or 2, respectively, this difference ranged from 14 to 23 days under moderate stress (**Table 1**).

For the severe stress (E3-SD), the 5d difference between genotypes was not significant and relatively small compared to moderately water-stressed plants in the other two experiments.

Overall, both genotypes had similar phenology under well-watered condition and severe water stress, but only Mace has a post-anthesis period significantly reduced by a moderate water stress.

### 3.2 Under well-watered conditions, dry root biomass at depth increased post anthesis in Scout but not in Mace

**Figure 1:**
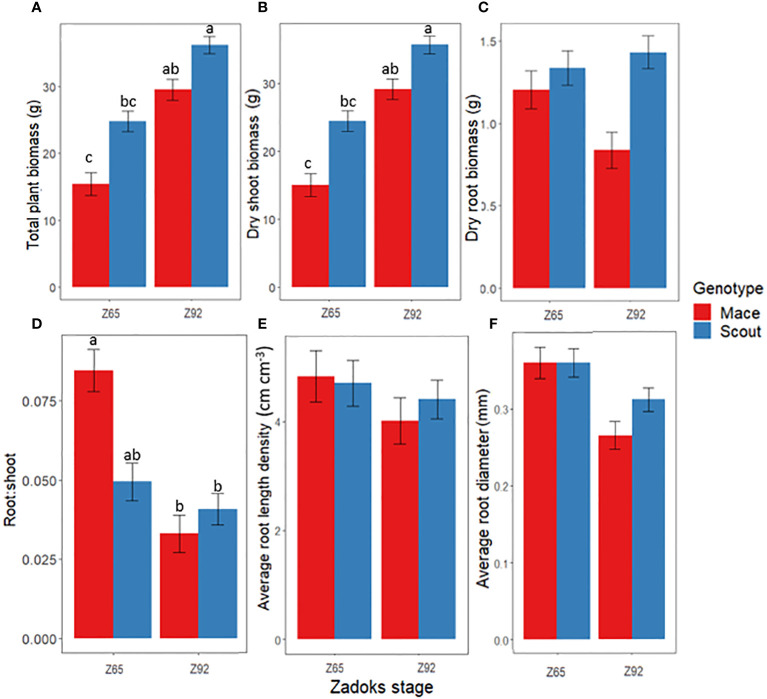
Changes between anthesis (Z65) and maturity (Z92) under well-watered conditions (E1-WW) in **(A)** whole-plant dry biomass, **(B)** shoot dry biomass, **(C)** root dry biomass, **(D)** root: shoot dry biomass ratio, **(E)** average root length density, and **(F)** average root diameter for Mace (red bars) and Scout (blue bars). Different letters indicate mean values that are significantly different at P<0.05. Error bars represent the standard error of the mean (n=8). The results from the analysis of variance associated to those data are presented in **Supplementary Table S1**.

Under well-watered conditions, whole-plant dry biomass significantly increased from anthesis to maturity for both Scout and Mace, but genotype x stage interactions were not significantly different (**Figure 1A**; **Supplementary Table S1**). Mace tended to have a smaller total plant biomass than Scout both at anthesis and maturity (**Figure 1A**) mainly due to a smaller shoot system. Root biomass only accounted for 2.5–10% of the shoot biomass (**Figures 1B–D**). Mace also tended to have lesser root biomass than Scout, especially at maturity (Z92; **Figure 1C**).

**Figure 2:**
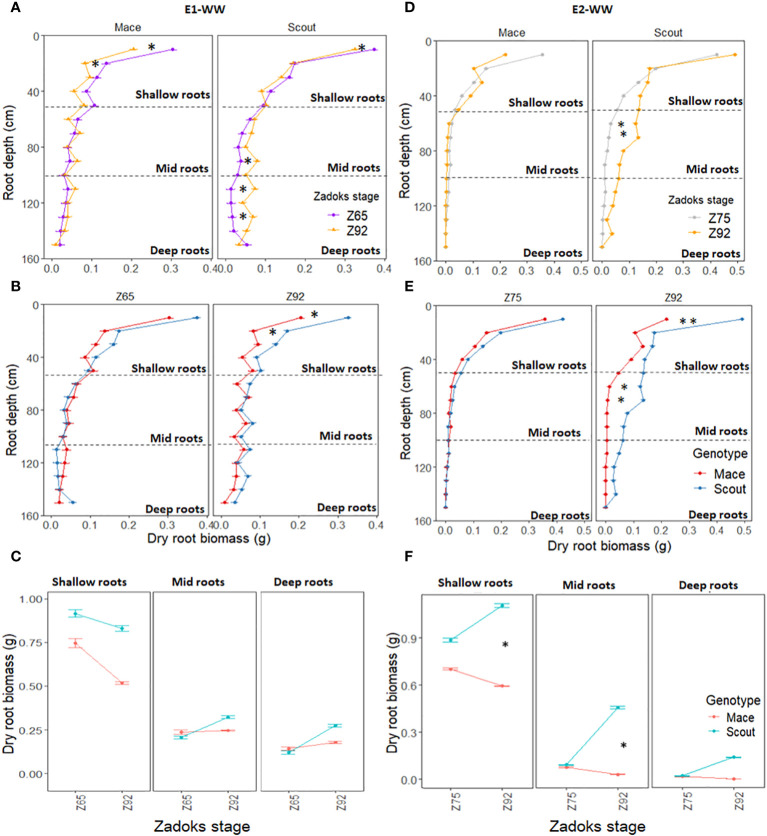
Dry root biomass at different depths (0 to 150 cm) for Mace and Scout plants grown under well-watered conditions in E1-WW **(A–C)** and E2-WW **(D–F)** and harvested at different stages, including anthesis (Z65), mid-grain filling (Z75) and maturity (Z92). In **(A, B, D, E)**, the horizontal dashed lines represent partitions between shallow (0 to 50 cm), mid (50 to 100 cm), and deep (100 to 150 cm) root layers. Error bars represent the standard error of the mean (n=8). Significant differences between means for root layers are indicated by asterisks for P<0.05* and P<0.01**.

Significant post-anthesis root growth occurred for roots deeper than 50 cm in Scout under well-watered conditions (**Figures 2A, D**), with a net increase in root biomass between anthesis and maturity (**Figures 2A, D**). In contrast, for Mace, root biomass changed little from anthesis and maturity at all depths deeper than 50 cm, while some reduction in root biomass was observed for the shallow soil layers (**Figures 2A, D**).

Overall, the post-anthesis increase in shoot biomass with little or no increase in root biomass led to a decrease in the root: shoot ratio from anthesis to maturity for both genotypes (**Figure 1D**).

**Figure 3:**
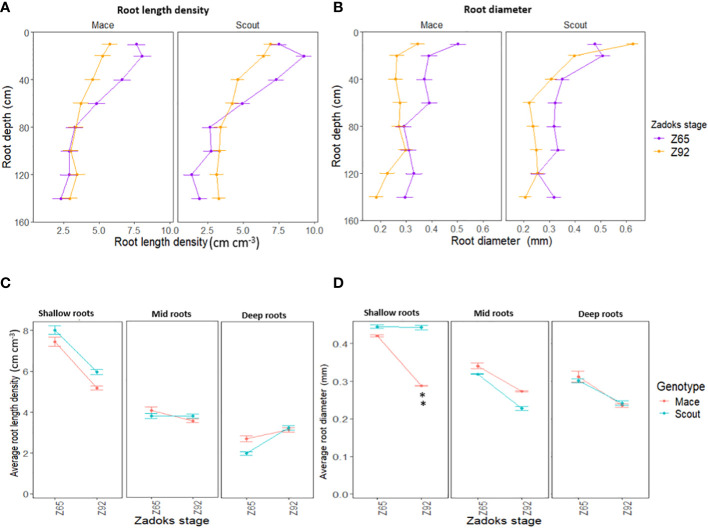
Average root length density **(A, C)** and root diameter **(B, D)** of Mace and Scout at anthesis (Z65) and maturity (Z92) under well-watered conditions (E1-WW) at different 10-cm depth intervals (0–10, 20–30, 50–60, …, 130–140 cm) **(A, B)**, and for corresponding shallow (0 to 50 cm), mid (50 to 100 cm) and deep (100 to 150 cm) roots **(C, D)**. Error bars represent the standard error of the mean (n=8). Asterisks (**) indicate significant genotypic difference at P<0.01.

Post-anthesis root length density tended to decrease in both genotypes (**Figure 1E**). This decrease was observed for shallow roots (<50 cm) in both genotypes, while post-anthesis root length density tended to increase in deep roots (> 50 cm) in Scout (**Figures 3A, C**).

For both genotypes, average root diameter tended to decrease after anthesis at all depths under well-watered conditions (**Figures 1F**, **3B, D**).

### 3.3 Moderate water stress reduced both shoot and root biomass in Mace, while Scout was more tolerant

**Figure 4:**
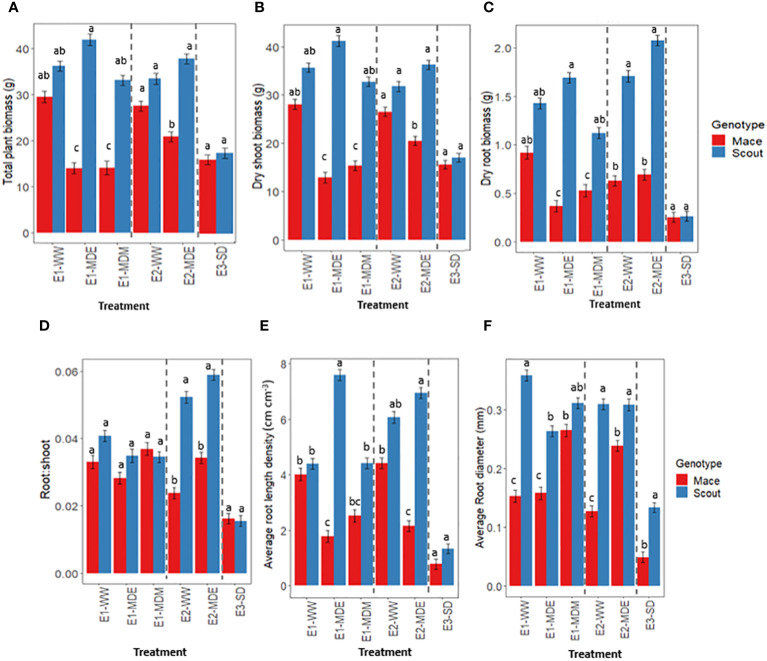
Response to different soil water status at maturity for **(A)** whole-plant dry biomass, **(B)** shoot dry biomass, **(C)** root dry biomass, **(D)** root: shoot dry biomass ratio, **(E)** average root length density, and **(F)** average root diameter in Mace (red bars) and Scout (blue bars). The names of the different treatments correspond to the experiment (E1, E2, E3) followed by “WW” for well-watered, “MDE” for moderate drought early in grain filling, “MDM” for moderate drought mid-grain filling or “SD” for severe drought with water being withheld from head emergence to maturity (**Table 1**). In each panel, dashed lines separate the data from the three experiments. Analysis of variance was performed separately for each experiment (**Supplementary Table S2**). Different letters indicate mean values that are significantly different at P<0.05 within each experiment. Error bars represent the standard error of the mean (n=8).

Total plant biomass at maturity in well-watered conditions was not significantly different between Scout and Mace although Mace tended to have a lower mean value (**Figure 4A**). In contrast, significant differences were observed between genotypes in moderately water-stressed treatments (**Figure 4A**). Moderate water-stress treatments during the early or mid-grain filling period significantly reduced whole-plant biomass at maturity in Mace, with a reduction by 48.8%, 37.6% and 32.78% for E1-MDE, E1-MDM, and E2-MDE, respectively, compared to their respective well-watered controls (E1-WW and E2-WW; **Figure 4A**). By contrast, whole-plant biomass of Scout was little affected by moderate water stress and remained similar to that observed in well-watered conditions. This distinction between genotypes was lost in the severe water stress treatment (E3-SD) in experiment E3, which substantially reduced the total plant biomass of both genotypes to similarly low values.

A similar trend was observed for dry shoot biomass with greater difference between the genotypes for moderate water stress treatments (E1-MDE, E1-MDM and E2-MDE) compared to either the well-watered or severe water stressed treatments (**Figure 4B**).

Root biomass and root length density also followed an overall similar trend (**Figures 4C, E**). Average root diameter was greater in Scout than in Mace in most treatments, including under well-watered conditions. By contrast, a moderate water stress had either no effect or increased the average root diameter in Mace, while it decreased average root diameter in Scout in experiment E1 (**Figure 4F**).

The root: shoot ratio at maturity did not vary significantly between treatments or between genotypes in experiment E1 (**Figure 4D**; **Supplementary Table S2**). However, in experiment E2, significant differences were observed between genotypes both in E2-WW and E2-MDE, with Scout having a greater root: shoot ratio than Mace (**Figure 4D**). For the severe water-stress treatment of experiment E3 (E3-SD), no significant difference was observed between Scout and Mace for root: shoot ratio. The mean values for root: shoot ratio, although they cannot be formally compared between experiments, were much lower in E3 than in any of the treatments in E1 and E2.

### 3.4 Moderate water stress induced root senescence at all depths in Mace, and in shallow roots in Scout

For the studied root traits, significative differences were found between genotypes, treatments, depths and their interactions (**Supplementary Table S3**).

**Figure 5:**
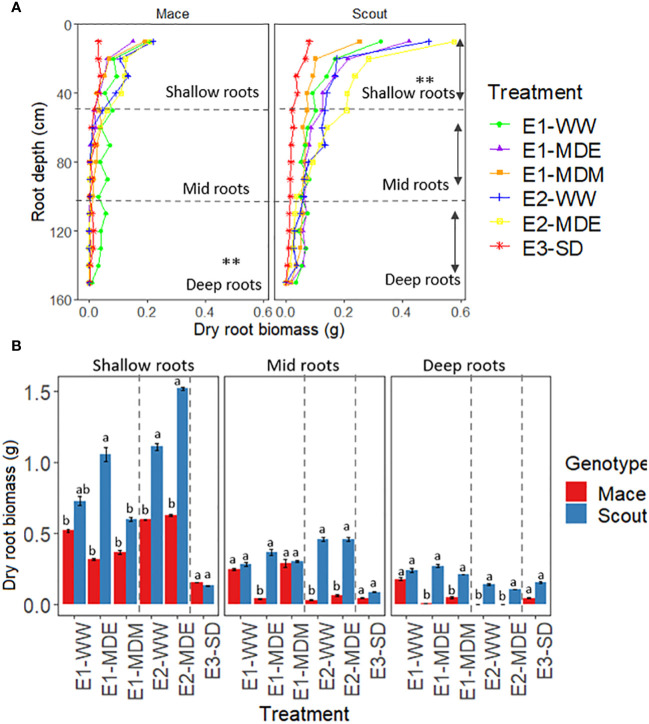
Distribution of dry root biomass per plant for different soil water treatments at maturity for **(A)** 10-cm increment depths (0 to 150 cm) and **(B)** shallow (0 to 50 cm), mid (50 to 100 cm), and deep (100 to 150 cm) roots in Mace and Scout. In **(A)**, asterisks (**) indicate differences between treatments for dry biomass of shallow, mid or deep roots (P<0.01). In **(B)**, the dotted lines separate the three independently analyzed experiments; means that are significantly different (P<0.05) within each experiment are indicated by different letters above the bars. Error bars represent the standard error of the mean (n=8). The results of analyses of variance from each experiment are presented in **Supplementary Table S3**.

For Mace, no significant difference was observed in root biomass between water treatments at shallow depths (0–50 cm), but moderate water stress (E1-MDE, E1-MDM and E2-MDE) caused root senescence for lower depths (100–150 cm) compared to well water treatments (E1-WW and E2-WW; **Figure 5**; **Supplementary Figure S1**). By contrast in Scout, no substantial difference in root biomass was observed for deep roots between well-watered (E1-WW and E2-WW) and moderate water stress treatments. However, shallow roots of Scout after a moderate stress during mid-grain filling (E1-MDM) or a severe stress (E3-SD) had less biomass than well-watered plants at maturity, indicating root senescence after anthesis. By contrast, for an earlier moderate stress during early grain filling (E1-MDE and E2-MDE), Scout shallow roots tended to grow more than under the other treatments, including well-watered conditions (E1-WW and E2-WW). In the severely water stressed treatment (E3-SD), dry root biomass for shallow and deep roots was similarly severely reduced for both genotypes at maturity.

**Figure 6:**
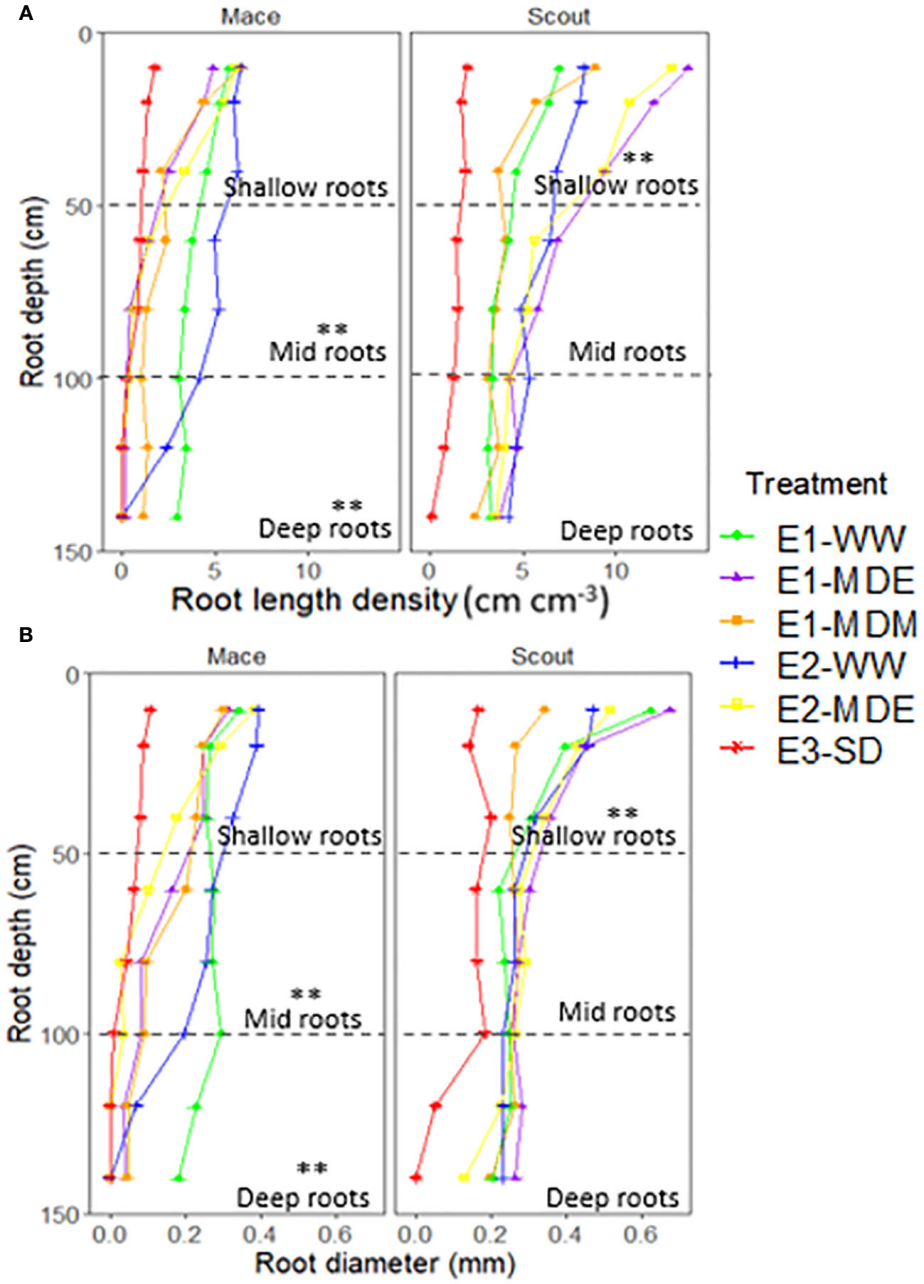
Root length density **(A)** and root diameter **(B)** of Mace and Scout for different depths (0 to 150 cm) at maturity for different water status treatments. The dashed lines represent partitions between shallow (0 to 50 cm), mid (50 to 100 cm), and deep (100 to 150 cm) roots. Asterisks (**) indicate differences between treatments for shallow, mid or deep roots (P<0.01). The results of analyses of variance from each experiment are presented in **Supplementary Table S3**.

Root length density and average root diameter for shallow and deep layers had a similar trend to that of root biomass under all treatments for both Scout and Mace (**Figure 6**; **Supplementary Figures S2**, **S3**).

**Figure 7:**
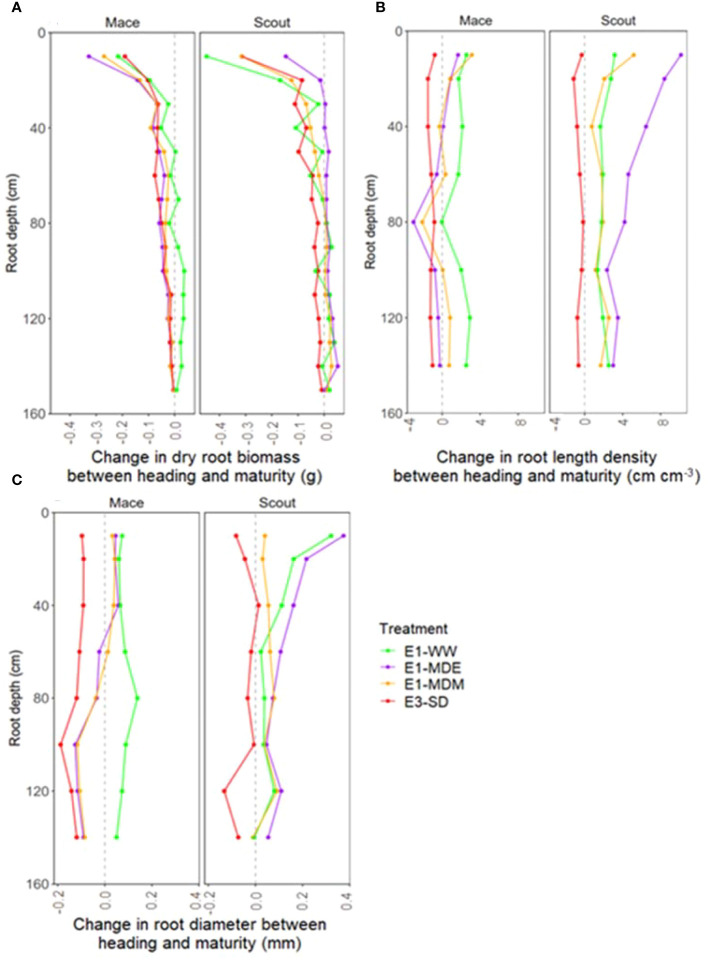
Change in whole-plant root biomass **(A)**, root length density **(B)**, and root diameter **(C)** between heading (Z50) and maturity (Z92) for Mace and Scout at different soil depths (0 to 150 cm) in all studied water treatments where measurements at such stages were performed (i.e. all treatments of experiments E1 and E3). In each panel, the vertical dashed grey line corresponds to a value of zero representing no change between stages. Values to the left of the line **(A)** represent a decrease in biomass (net root senescence) while values to the right represent increase in biomass (net root growth).

To investigate dynamic changes in root development, variations in dry root biomass distribution between heading (Z50) and maturity (Z92) were estimated by subtracting the root biomass at heading from the root biomass at maturity (**Figure 7A**; **Supplementary Figure S4**), complementing comparison previously done between anthesis and maturity (e.g., **Figures 1**, **2A–C**, **3**). For Mace, all water-stress treatments tended to cause net shallow and deep root senescence between heading and maturity, while in well-watered conditions, net senescence was only observed in shallow roots and to a lesser extent than for stressed conditions (**Figure 7A**). In contrast for Scout, deep root biomass increased between heading and maturity in all treatments, except for the severe water-stress treatment (E3-SD) where net senescence was observed. For Scout shallow roots, senescence was observed for all treatments except E1-MDE. In other words, deep roots for Scout grew between heading and maturity in all studied conditions, except the severe stress E3-SD which induced a net root senescence in all soil layers.

Root length density in Scout was greater at maturity than at heading for all depths in all treatments except E3-SD where a small net senescence occurred (**Figure 7B**). Roots of Scout also increased in diameter on average between heading and maturity for all treatments except E3-SD (**Figure 7C**). For Mace, shallow and deep layer root length density between heading and maturity was reduced by water stress. Mace average root diameter was also reduced by water stress, except for shallow roots under moderate water stress which had a similar root diameter as under well-watered conditions (**Figure 7C**; **Supplementary Figure 3C**). The differences in root diameter between Scout and Mace were accentuated by all water stress treatments.

### 3.5 Water stress induced more canopy senescence in Mace than Scout

**Figure 8:**
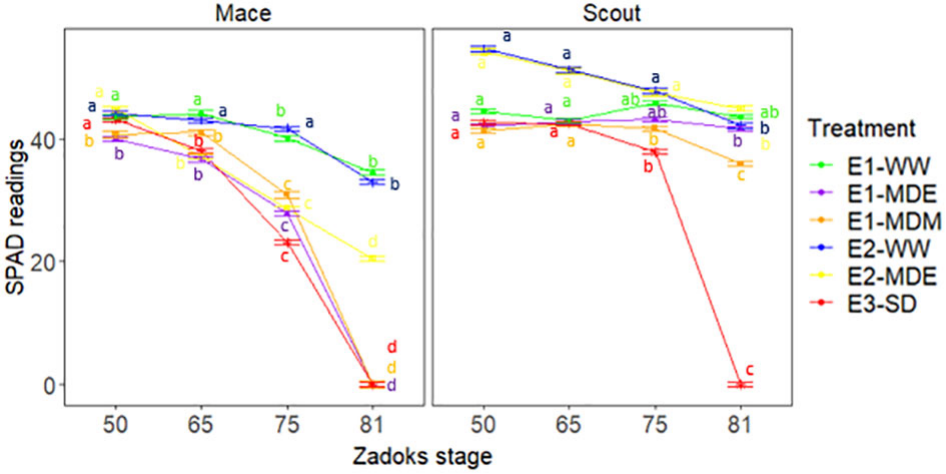
Dynamics of leaf greenness as indicated by SPAD values of Mace and Scout in all studied water treatments. SPAD values were measured in the center of the flag leaf. Means that are significantly different (P<0.05) between Scout and Mace within each experiment are shown by the same letters above points. The analysis of variance was done separately for each experiment (**Supplementary Table S4**). Error bars represent the standard error of the mean (n=8).

**Figure 9:**
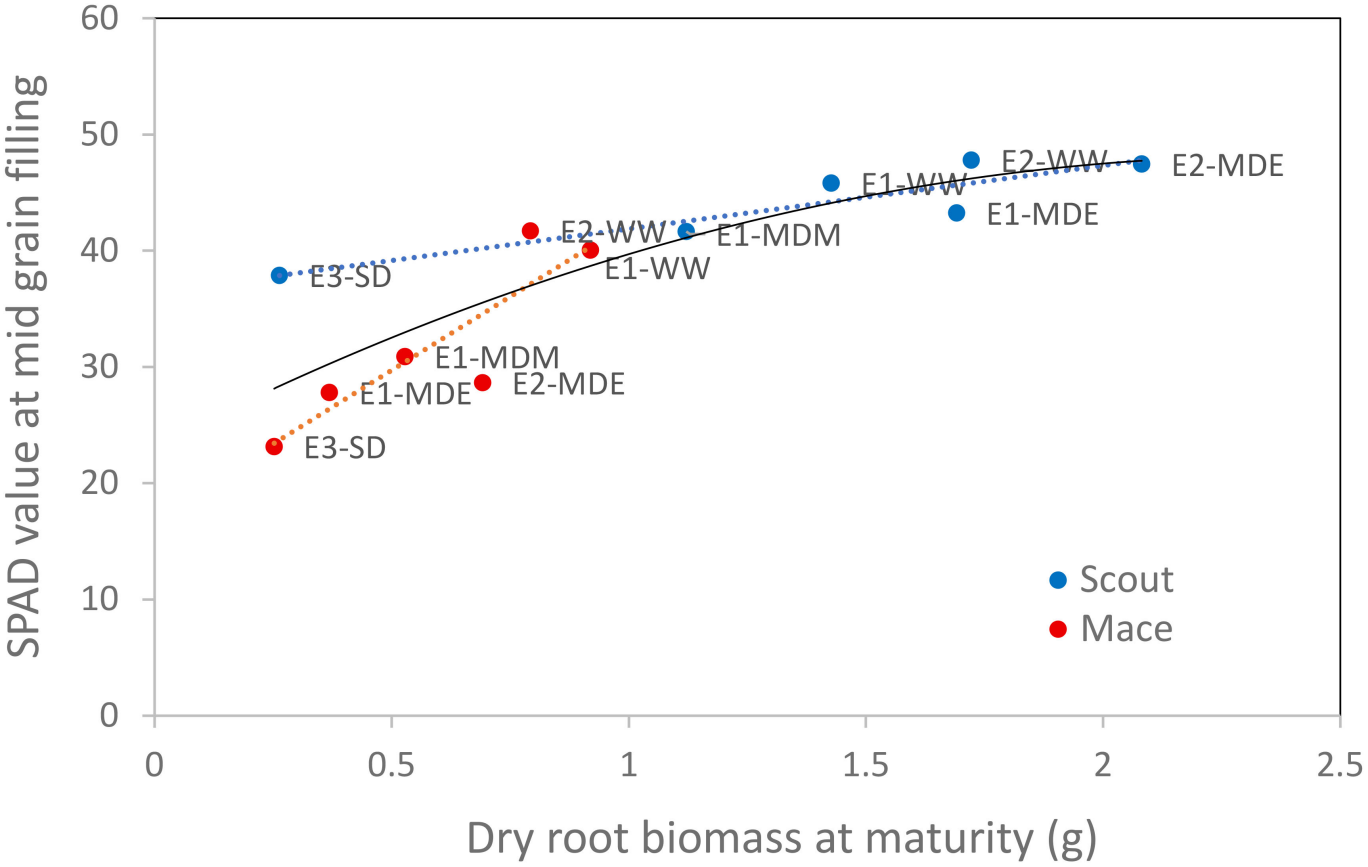
Relationship between leaf greenness (SPAD value) at mid grain filling (Z75) and the whole-plant dry root biomass at maturity (Z92) for both Mace and Scout in all studied water treatments. Linear regressions are presented for each genotype (dotted red line for Mace, r^2^ of 0.78; dotted blue line for Scout, r^2^ of 0.82), as well as a polynomial regression fitted on all data (solid black line, r^2^ of 0.72).

To address the question of whether root senescence observed for some water-stress treatments was related to canopy senescence, the greenness of the flag leaf was followed post anthesis using SPAD measurements (**Figures 8**, **9**). Significative differences were found for SPAD values between genotypes, stages, treatments, and their interactions (**Supplementary Table S4**).

Under well-watered conditions (E1-WW and E2-WW), both cultivars exhibited relatively high leaf SPAD values greater than 38 arbitrary SPAD units until late grain filling (Z81). In E1-WW, Scout had higher chlorophyll content than Mace at heading and anthesis and exhibited a small drop over the grain filling period, which was also observed in Mace (**Figure 8**). Leaf area was also significantly higher for Scout than Mace at heading (Z50) under well-watered conditions (**Table 1**).

Moderate water stress (E1-MDE, E1-MDM, E2-MDE) resulted in accelerated leaf senescence during the grain filling (e.g., Z75) in Mace, while there was little or no effect on Scout flag leaf greenness (**Figure 8**). Severe water stress (E3-SD) caused a rapid decrease in leaf greenness for both cultivars after anthesis (Z65) although Scout had significantly higher mean SPAD values than Mace up until mid-grain filling (Z75). Although not formally comparable, the mean leaf greenness (SPAD) for Scout at late grain filling (Z81) for E3-SD were reduced to a lower value than for any of the treatments in either experiment E1 or E2 for this genotype.

Mace showed leaf senescence from anthesis (Z65) to maturity (**Figure 8**) which was correlated with loss of deep root biomass between heading (Z50) and maturity (**Figure 7**) under moderate and severe stress. Similarly, leaf senescence in Scout correlated with root senescence in severe stress, while little or no senescence was observed in either deep roots or shoots of Scout under moderate stress (**Figures 7**, **8**).

A strong correlation was found between root dry biomass at maturity (which results from root growth and senescence that occurring during the whole plant cycle) and leaf greenness (SPAD) at mid grain filling (**Figure 9**).

### 3.6 Water stress induced more yield loss in Mace than Scout

In well-watered conditions, the grain yield per plant in Mace (16 ± 1.5 and 12 ± 0.4 g in E1-WW and E2-WW, respectively) was similar to that of Scout (14 ± 1.5 and 13 ± 0.5 g; **Table 1**).

For moderate water stress, the yield per plant in Scout in treatments E1-MDE (16 ± 1.8 g), E1-MDM (12 ± 1.5 g) and E2-MDE (14 ± 0.4 g) remained similar to that in well-watered plants. In contrast, for Mace, the yield per plant was significantly lower for E1-MDE (7 ± 1.6 g), E2-MDE (8 ± 0.4 g), and E1-MDM (8 ± 1.8 g) compared with E1-WW and E2-WW, respectively (**Table 1**).

In severe water stress (E3-SD), yield of both genotypes was severely reduced compared with E1-WW or E2-WW with similarly low values of 2.5 ± 0.19 g for Mace and 3 ± 0.19 g for Scout (**Table 1**).

Overall, while Mace and Scout had similar grain yield per plant when well watered, only Scout maintained it under moderate drought. Severe water stress severely affected yield of both genotypes.

## 4 Discussion

The polyvinyl chloride (PVC) pot system was successfully used to identify genotypic differences in the response of wheat roots to drought. Two wheat cultivars known to differ in seedling seminal root angle were used to demonstrate this (Richard, 2017; Richard et al., 2018).

### 4.1 Moderate water stress induced root senescence or root growth depending on the genotype

In this study, Scout had similar or significantly greater shallow and deep root biomass than Mace at maturity (**Figures 2**, **5**; **Supplementary Figure S1**). Deep root biomass of Scout (i) significantly increased after anthesis under well-watered conditions (**Figures 2A, C**), (ii) tended to slightly increase between heading and maturity when under moderate water stress (**Figure 5**), but (iii) decreased post-heading in severe water-limited conditions (**Figures 3**, **7**; **Supplementary Figure S4A**). By contrast, shallow root biomass of Scout (i) senesced post-heading under well-watered, mid-grain filling moderate water-stress and severe water-stress conditions and (ii) tended to have a similar biomass at heading and maturity under early-grain-filling water stress, thus indicating some stress-induced root growth in these conditions. Overall, under water deficit conditions, Scout maintained deep roots more than shallow roots (**Figure 7**).

Conversely, Mace did not have post-anthesis deep root growth under well-watered conditions (**Figure 2**), showed post-anthesis root senescence of shallow roots under well-watered conditions (**Figure 2**), and showed shallow and deep root senescence for moderate and severe post-anthesis water stress treatments (**Figure 7**). Notably, Mace had a slightly smaller green leaf area than Scout at anthesis (**Table 1**) and experienced earlier leaf senescence (**Figure 8**), indicating that Mace may be less prone to soil water loss by transpiration due to its slightly smaller green leaf area (**Table 1**). However, Mace may also have been more prone to water loss, due to a higher hydraulic conductance suggested by observations of greater normalized transpiration rate at high evaporative demand (Chenu et al. unpublished) and lower transpiration efficiency (Fletcher et al., 2018) in Mace than Scout. Overall, in the present study, Mace appeared more susceptible to the imposed water stress conditions than Scout in terms of root development, which is likely to at least partly explain the greater yield reductions observed in Mace compared to Scout. The greater plasticity of Mace in response to late water stress was also observed in a field trial at Hermitage, Queensland, where Mace yield was reduced by 15% in the rainfed treatment, while Scout maintained its yield under these conditions (Christopher et al., unpublished).

Despite these results, Mace is reputed to be well-adapted for other drought-prone regions, such as the Southern and Western parts of the Australian wheatbelt. Mace has been widely grown in these regions (Ehdaie et al., 2012). Mace was also observed to produce a similar or greater yield than Scout in field trials in these regions (Christopher et al., unpublished). Large parts of those regions have a Mediterranean climate characterized by winter-dominant rainfall (Singh et al., 2011) with medium to light soils, in which a quick finish to avoid terminal drought and heat may be more advantageous than developing post-anthesis root growth to extend the grain filling period (Shavrukov et al., 2017). Such traits were observed in Mace under stress in the current study (**Table 1**). The wide root angle observed at the seedling stage in Mace (Richard et al., 2018) may also confer some benefit for shallow root development in field conditions to better forage for water from small rainfall events leading to only shallow moisture infiltration. It is noteworthy that in the long PVC pot, roots were constrained laterally (90 mm diameter), which may have affected the development of shallow roots. In this system, roots could for example choose the path of least resistance down the side of the soil column. For instance, it has been observed that roots have a propensity to develop in pores at depth in the field (White and Kirkegaard, 2010; Hodgkinson et al., 2017). However, in the current experiments, the soil was well watered up until heading, and later in most conditions, and any strinkage of soil leading to gaps at the sides of the columns would likely have occurred too late in the crop cycle to have a major effect on root accumulation at the edges. Also, the root: shoot ratios observed in the current study were relatively low compared to some other studies, but they were in a similar order to post-anthesis root: shoot ratios estimated based on soil cores in irrigated wheat field trials (Lopes and Reynolds, 2011; Heinemann et al., 2023).

### 4.2 Deep rooting as a drought adaptative trait associated with stay-green, increased crop cycle duration and enhanced yield

The narrow seedling root angle previously reported for Scout has been associated for other wheat genotypes exhibiting development of deep roots and the ability to access water stored deep in the soil (Ludlow and Muchow, 1990). In agreement with this, in the current study, Scout also had a greater root biomass and root length density at depth than Mace late in the season, especially under moderate water stress (**Figure 5**; **Supplementary Figure S1**). However, the severe prolonged water-stress treatment caused both root and leaf senescence for the two genotypes, as plants cannot survive without access to water (Vadez et al., 2024). The greater deep root biomass and root length density observed for Scout post-anthesis, especially following moderate late water stress (e.g., **Supplementary Figures S1**-**S2**), is likely to have allowed Scott to access more water at depth than Mace, although water uptake was not directly measured. However, the observed stay-green phenotype with plants retaining a higher green leaf area content until close to maturity tend to support the notion that Scout could access water for longer in the season (**Figures 8**, **9**). Such a stay-green phenotype can allow plants to accumulate biomass for longer and to mature later than plants lacking the stay-green phenotype, helping to explain the greater duration to maturity time observed in Scout versus Mace under water stress (**Table 1**). Concordantly, in field study, the stay-green phenotype with retention of both green leaf area and photosynthetic capacity for longer during grain filling, has been associated with higher yield for several plant species (Thomas and Smart, 1993; Nawaz et al., 2013; Christopher et al., 2014). This is in particular the case under post-anthesis drought stress for bread wheat (Christopher et al., 2008; Adu et al., 2011; Bogard et al., 2011; Lopes and Reynolds, 2012), durum wheat (Spano et al., 2003), and other cereals such as maize (Crafts-Brandner et al., 1984; Gentinetta et al., 1986; Zheng et al., 2009), rice (Mondal et al., 1985; Wada and Wada, 1991; Ba Hoang and Kobata, 2009), and sorghum (Borrell et al., 2014 and Borrell et al., 2023).

### 4.3 Implication for plant breeding

High levels of variation in root traits, and possible emergent traits such as stay-green and canopy temperature have been reported across large populations in different crops including wheat (Christopher et al., 2014; Richard et al., 2015 and Lopes and Reynolds, 2010; Christopher et al., 2016). However, the value of root traits for crop improvement in different target populations of environments (TPE) remains vaguely defined. Crop modelling studies have been conducted in an attempt to assess these values (e.g. MansChadi et al., 2006; Veyradier et al., 2013; Casadebaig et al., 2016; Lilley and Kirkegaard, 2016), but with models that are not well adapted to simulate root growth and development, especially not across genotypes. The present study is a step forwards to characterize the dynamics of root architecture in response to environments, as demonstrated by the contrast between the two genotypes studied here. This, and further such studies can help to improve our modelling capability for the effects of root traits in various environments (Chenu et al., 2017).

Genetics studies have also examined correlations between root traits with yield as well as the co-location of QTL for root traits and yield. In sorghum, QTL for nodal root angle at the seedling stage were found to co-locate with QTL for traits associated with drought adaptation (Mace et al., 2012), indicating the potential value of this trait. In barley, correlation between seedling root traits and yield were highly dependent on the environment considered (Robinson et al., 2018). The relationship between QTL for seminal root traits and yield was less clear in bread wheat (Christopher et al., 2021).

To advance in this field, phenotyping methods such as the one presented here should be adapted to be able to screen large populations for root characteristics at important stages such as during the grain filling. In the field, methods using minirhizotrons or soil cores associated for instance with fluorescence spectroscopy exist to look at deep rooting (Wasson et al., 2016; Christopher, 2024). Recently, a field-based method based on electro-magnetic induction (EMI) sensors (DualEM 21S) was proposed to screen for changes in soil water at different depths to indirectly characterize root systems at depth (Zhao et al., 2022). This non-destructive method was successfully applied to investigate differences between sorghum hybrids (Zhao et al., 2023; Zhao et al., 2024) and could potentially be extended to investigate root plasticity to water stress and to identify underlying genetic controls in different crops including wheat.

## 5 Conclusion

The results of this study suggest that Scout maintained post-anthesis deep root growth under moderate post-anthesis water-stress treatment whereas deep roots of Mace senesced. Deep root development potentially enabled access to water late in the season which likely aided Scout to maintain a stay-green phenotype and produce higher yield per plant under moderated water stress. The reported findings based on two contrasting genotypes could be extended to a greater range genotypes.

The results have implications for crop modelling which does not yet adequately consider root growth, development and water-and-nutrient uptake.

Identification of root traits that may enhance water uptake late in crop development, such as deep root length density for drought-prone locations with heavy deep soils, is anticipated to improve wheat adaptation in regions prone to late season water stress.

## Data availability statement

The raw data supporting the conclusions of this article will be made available by the authors, without undue reservation.

## Author contributions

KS: Conceptualization, Data curation, Formal analysis, Investigation, Methodology, Visualization, Writing – original draft. JC: Conceptualization, Investigation, Methodology, Resources, Supervision, Writing – review & editing. KC: Conceptualization, Data curation, Investigation, Methodology, Resources, Supervision, Writing – review & editing.
