# Supplementary material for: Does late water deficit induce root growth or senescence in wheat?
Source: Front Plant Sci. 2024 Jun 7;15:1351436. doi: 10.3389/fpls.2024.1351436 (PMC11190305; doi:10.3389/fpls.2024.1351436)
Supplement: Supplementary file 1 [file DataSheet_1.docx]

Supplementary Material

Does late water deficit induce root growth or senescence in wheat?

Kanwal Shazadi, John T. Christopher, Karine Chenu*

*** Correspondence:** Karine Chenu [karine.chenu@uq.edu.au](mailto:karine.chenu@uq.edu.au)

# Supplementary Tables

**Supplementary Table S1.** Analysis of variance for shoot and root traits at anthesis (Z65) and maturity (Z92) developmental stages in the well-watered treatment of Experiment 1 (E1-WW). Significant differences are indicated by asterisks for P<0.05*, P<0.01**, and P<0.001***. A bar chart of those traits at anthesis and maturity is presented for Mace and Scout in Figure 1.

|  |  | **Experiment 1 (E1-WW)** | | |
| --- | --- | --- | --- | --- |
|  |  | Df | F Value | Pr(>F) |
| **Total plant biomass (g)** | |  |  |  |
|  | Genotype | 1 | 7.32 | 0.0150 * |
|  | Stage | 1 | 17.17 | 0.0007 *** |
|  | Genotype: Stage | 1 | 0.19 | 0.6649 |
| **Total dry shoot biomass (g)** | | |  |  |
|  | Genotype | 1 | 7.20 | 0.0157 * |
|  | Stage | 1 | 17.03 | 0.0007 *** |
|  | Genotype: Stage | 1 | 0.22 | 0.6433 |
| **Total dry root biomass (g)** | | |  |  |
|  | Genotype | 1 | 3.18 | 0.0922 |
|  | Stage | 1 | 0.01 | 0.9209 |
|  | Genotype: Stage | 1 | 0.95 | 0.3431 |
| **Root: shoot ratio** | |  |  |  |
|  | Genotype | 1 | 0.97 | 0.3383 |
|  | Stage | 1 | 5.37 | 0.0332 * |
|  | Genotype: Stage | 1 | 3.30 | 0.0872 |
| **Average root length density (cm cm^-3^)** | | | |  |
|  | Genotype | 1 | 0.03 | 0.8553 |
|  | Stage | 1 | 0.39 | 0.5419 |
|  | Genotype: Stage | 1 | 0.09 | 0.7630 |
| **Average root diameter (mm)** | | |  |  |
|  | Genotype | 1 | 0.47 | 0.50014 |
|  | Stage | 1 | 3.69 | 0.07168 |
|  | Genotype: Stage | 1 | 0.42 | 0.52624 |

**Supplementary Table S2.** Analysis of variance for shoot and root traits at maturity (Z92) in each experiment. Significant differences are indicated by asterisks for P<0.05*, P<0.01**, and P<0.001***. A bar chart of the genotypic responses from those traits to the different water treatments is presented in Figure 4.

|  |  | **Experiment 1** | | |  | **Experiment 2** | | |  | **Experiment 3** | | |
| --- | --- | --- | --- | --- | --- | --- | --- | --- | --- | --- | --- | --- |
|  |  | Df | F Value | Pr(>F) |  | Df | F Value | Pr(>F) |  | Df | F Value | Pr(>F) |
| **Total plant biomass (g)** | |  |  |  |  |  |  |  |  |  |  |  |
|  | Genotype | 1 | 45.04 | 2.76e-07 *** |  | 1 | 240.72 | 1.125e-13 *** |  | 1 | 8.85 | 0.01005 * |
|  | Treatment | 2 | 3.39 | 0.04795 * |  | 1 | 3.54 | 0.07256 |  |  |  |  |
|  | Genotype: Treatment | 2 | 5.72 | 0.00827 ** |  | 1 | 54.40 | 1.679e-07 *** |  |  |  |  |
| **Dry shoot biomass (g)** | |  |  |  |  |  |  |  |  |  |  |  |
|  | Genotype | 1 | 47.67 | 0.0000001 *** |  | 1 | 196.23 | 9.506e-13 *** |  | 1 | 8.79 | 0.01025 * |
|  | Treatment | 2 | 3.49 | 0.04336 * |  | 1 | 1.68 | 0.2083 |  |  |  |  |
|  | Genotype: Treatment | 2 | 5.37 | 0.01018 * |  | 1 | 46.73 | 5.701e-07 *** |  |  |  |  |
| **Dry root biomass (g)** | |  |  |  |  |  |  |  |  |  |  |  |
|  | Genotype | 1 | 36.42 | 0.000001 *** |  | 1 | 452.93 | < 2.2e-16 *** |  | 1 | 0.57 | 0.4641 |
|  | Treatment | 2 | 2.20 | 0.127 |  | 1 | 12.71 | 0.001642 ** |  |  |  |  |
|  | Genotype: Treatment | 2 | 3.71 | 0.036 * |  | 1 | 6.78 | 0.015905 * |  |  |  |  |
| **Root: shoot ratio** | |  |  |  |  |  |  |  |  |  |  |  |
|  | Genotype | 1 | 1.31 | 0.2614 |  | 1 | 111.41 | 2.73e-10 *** |  | 1 | 0.46 | 0.51 |
|  | Treatment | 2 | 0.72 | 0.4939 |  | 1 | 11.55 | 0.00247 ** |  |  |  |  |
|  | Genotype: Treatment | 2 | 0.67 | 0.5196 |  | 1 | 0.59 | 0.4508 |  |  |  |  |
| **Average root length density (cm cm^-3^)** | | | |  |  |  |  |  |  |  |  |  |
|  | Genotype | 1 | 33.98 | 2e-06 *** |  | 1 | 195.12 | 1.01e-12 *** |  | 1 | 23.50 | 0.00026 *** |
|  | Treatment | 2 | 3.10 | 0.05906 |  | 1 | 10.27 | 0.00393 ** |  |  |  |  |
|  | Genotype: Treatment | 2 | 11.96 | 0.00014 *** |  | 1 | 44.76 | 7.97e-07 *** |  |  |  |  |
| **Average root diameter (mm)** | | | |  |  |  |  |  |  |  |  |  |
|  | Genotype | 1 | 32.78 | 2.687e-06 *** |  | 1 | 141.97 | 2.547e-11 *** |  | 1 | 20.90 | 0.00044 *** |
|  | Treatment | 2 | 4.01 | 0.02825 * |  | 1 | 28.82 | 1.878e-05 *** |  |  |  |  |
|  | Genotype: Treatment | 2 | 4.85 | 0.01473 * |  | 1 | 27.96 | 2.292e-05 *** |  |  |  |  |

**Supplementary Table S3.** Analysis of variance for root traits at maturity (Z92) for all treatments and depths in each of the three experiments. Significant differences are indicated by asterisks for P<0.05*, P<0.01**, and P<0.001***. Variations of those traits across different depths are presented for Mace and Scout in all treatments in Figures 5A and 6.

|  |  | **Experiment 1** | | |  | **Experiment 2** | | |  | **Experiment 3** | | |
| --- | --- | --- | --- | --- | --- | --- | --- | --- | --- | --- | --- | --- |
|  |  | Df | F Value | Pr(>F) |  | Df | F Value | Pr(>F) |  | Df | F Value | Pr(>F) |
| **Dry root biomass (g)** | |  |  |  |  |  |  |  |  |  |  |  |
|  | Genotype | 1 | 165.70 | < 2e-16 *** |  | 1 | 361.95 | < 2.2e-16 *** |  | 1 | 53.20 | 6.068e-12 *** |
|  | Treatment | 2 | 11.39 | 1.49e-05 *** |  | 1 | 10.16 | 0.00157 ** |  |  |  |  |
|  | Depth | 14 | 57.00 | < 2e-16 *** |  | 14 | 134.28 | < 2.2e-16 *** |  | 14 | 55.41 | < 2.2e-16 *** |
|  | Genotype: Treatment | 2 | 17.81 | 3.51e-08 *** |  | 1 | 5.41 | 0.0205571 * |  |  |  |  |
|  | Genotype: Depth | 14 | 3.78 | 4.57e-06 *** |  | 14 | 20.89 | < 2.2e-16 *** |  | 14 | 11.74 | < 2.2e-16 *** |
|  | Treatment: Depth | 28 | 0.91 | 0.606 |  | 14 | 1.94 | 0.02207 * |  |  |  |  |
|  | Genotype: Treatment: Depth | 28 | 1.07 | 0.367 |  | 14 | 1.64 | 0.06718 |  |  |  |  |
| **Average root length density (cm cm^-3^)** | | | |  |  |  |  |  |  |  |  |  |
|  | Genotype | 1 | 123.10 | < 2.2e-16 *** |  | 1 | 259.95 | < 2.2e-16 *** |  | 1 | 33.50 | 3.199e-08 *** |
|  | Treatment | 2 | 11.24 | 2.116e-05 *** |  | 1 | 13.69 | 0.00029 *** |  |  |  |  |
|  | Depth | 7 | 34.83 | < 2.2e-16 *** |  | 7 | 68.47 | < 2.2e-16 *** |  | 7 | 5.95 | 3.190e-06 *** |
|  | Genotype: Treatment | 2 | 43.31 | < 2.2e-16 *** |  | 1 | 59.63 | 7.096e-13 *** |  |  |  |  |
|  | Genotype: Depth | 7 | 1.21 | 0.30025 |  | 7 | 1.93 | 0.06710 |  | 7 | 2.93 | 0.00630 ** |
|  | Treatment: Depth | 14 | 2.58 | 0.00175 ** |  | 7 | 8.59 | 4.205e-09 *** |  |  |  |  |
|  | Genotype: Treatment: Depth | 14 | 0.73 | 0.73862 |  | 7 | 3.16 | 0.00350 ** |  |  |  |  |
| **Average root diameter (mm)** | | |  |  |  |  |  |  |  |  |  |  |
|  | Genotype | 1 | 87.27 | < 2.2e-16 *** |  | 1 | 92.93 | < 2.2e-16 *** |  | 1 | 74.58 | 4.548e-14 *** |
|  | Treatment | 2 | 11.14 | 2.337e-05 *** |  | 1 | 18.87 | 2.313e-05 *** |  |  |  |  |
|  | Depth | 7 | 25.78 | < 2.2e-16 *** |  | 7 | 44.24 | < 2.2e-16 *** |  | 7 | 13.56 | 1.361e-12 *** |
|  | Genotype: Treatment | 2 | 12.80 | 5.158e-06 *** |  | 1 | 18.30 | 3.027e-05 *** |  |  |  |  |
|  | Genotype: Depth | 7 | 2.50 | 0.01683 * |  | 7 | 1.31 | 0.24684 |  | 7 | 3.79 | 0.00100 *** |
|  | Treatment: Depth | 14 | 1.59 | 0.08233 |  | 7 | 0.94 | 0.47594 |  |  |  |  |
|  | Genotype: Treatment: Depth | 14 | 1.86 | 0.03164 * |  | 7 | 2.33 | 0.02687 * |  |  |  |  |

**Supplementary Table S4.** Analysis of variance for SPAD readings at different growth stages and treatments. Significant differences are indicated by asterisks for P<0.05*, P<0.01**, and P<0.001***. The dynamics of SPAD value for all genotypes and treatments is presented in Figure 8.

|  |  | **Experiment 1** | | |  | **Experiment 2** | | |  | **Experiment 3** | | | | |
| --- | --- | --- | --- | --- | --- | --- | --- | --- | --- | --- | --- | --- | --- | --- |
|  |  | Df | F Value | Pr(>F) |  | Df | F Value | Pr(>F) |  | Df | F Value | | Pr(>F) | |
| **SPAD values** | |  |  |  |  |  |  |  |  |  |  | |  | |
|  | Genotype | 1 | 1209 | <2e-16 *** |  | 1 | 563 | < 2.2e-16 *** |  | 1 | 325 | < 2.2e-16 *** | |  |
|  | Stage | 3 | 420 | <2e-16 *** |  | 3 | 146 | < 2.2e-16 *** |  | 3 | 4266 | < 2.2e-16 *** | |  |
|  | Treatment | 2 | 241 | <2e-16 *** |  | 1 | 61 | 1.383e-11 *** |  |  |  |  | |  |
|  | Genotype: Stage | 3 | 420 | <2e-16 *** |  | 3 | 8 | 0.00010 *** |  | 3 | 134 | < 2.2e-16 *** | |  |
|  | Genotype: Treatment | 2 | 147 | <2e-16 *** |  | 1 | 59 | 2.880e-11 *** |  |  |  | |  | |
|  | Stage: Treatment | 6 | 54 | <2e-16 *** |  | 3 | 10 | 1.234e-05 *** |  |  |  | |  | |
|  | Genotype: Stage: Treatment | 6 | 50 | <2e-16 *** |  | 3 | 12 | 1.297e-06 *** |  |  |  | |  | |

# Supplementary Figures


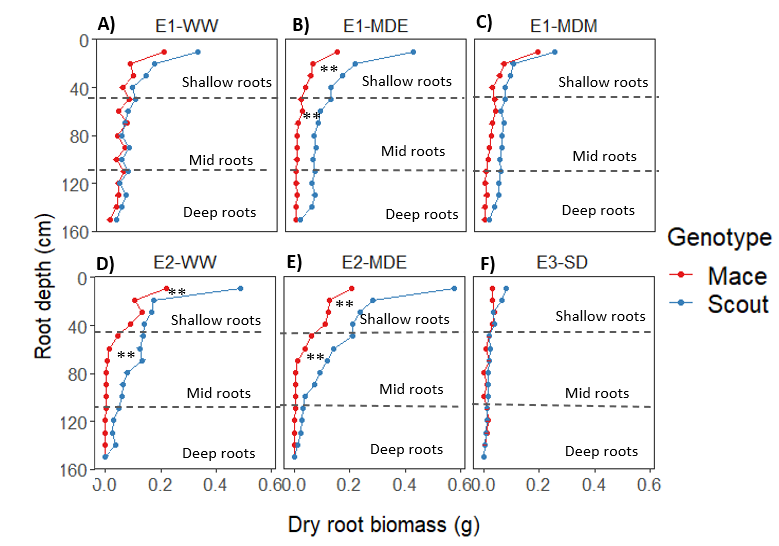


**Supplementary Figure S1.** Dry root biomass at different soil depths (0 to 150 cm) in Mace and Scout at maturity for the different studied water treatments. Panels (A, B and C) correspond to water stress treatments in E1; (D, E) water-stress treatments in E2; and (F) sever water stress in E3. The analysis of variance was performed separately for each treatment. Asterisks indicate genotypic differences for shallow (0 to 50 cm), mid (50 to 100 cm) or deep (100 to 150 cm) roots (P<0.01). This figure presents data from Figure 5A treatment by treatment.


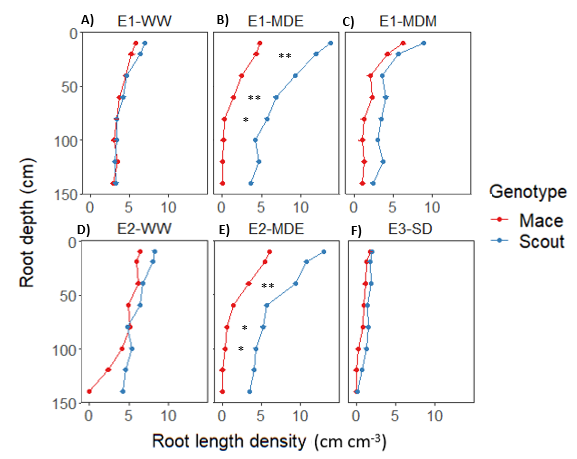


**Supplementary Figure S2.** Root length density at different soil depths in Mace and Scout at maturity for the different studied water treatments. Panels (A, B and C) correspond to water stress treatments in E1; (D, E) water-stress treatments in E2; and (F) sever water stress in E3. The analysis of variance was performed separately for each treatment. Asterisks indicate genotypic differences for roots at a specific depth (P<0.01). This figure presents data from Figure 6A treatment by treatment.


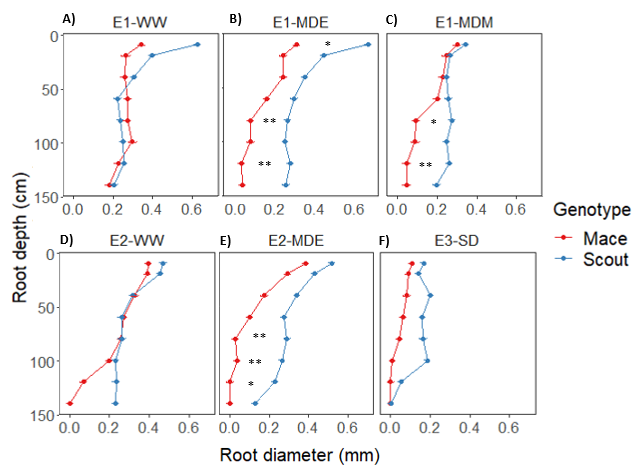


**Supplementary Figure S3.** Average root diameter at different soil depths in Mace and Scout at maturity for the different studied water treatments. Panels (A, B and C) correspond to water stress treatments in E1; (D, E) water-stress treatments in E2; and (F) sever water stress in E3. The Analysis of variance was performed separately for each treatment. Asterisks indicate genotypic differences for roots at a specific depth (P<0.01). This figure presents data from Figure 6B treatment by treatment.


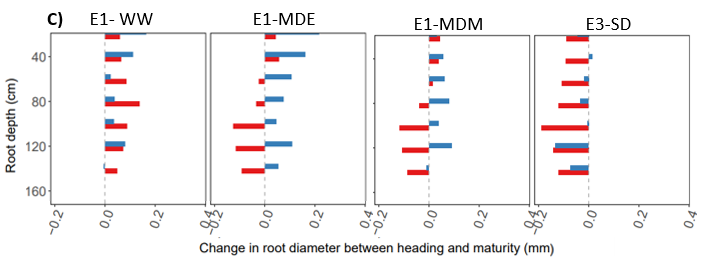

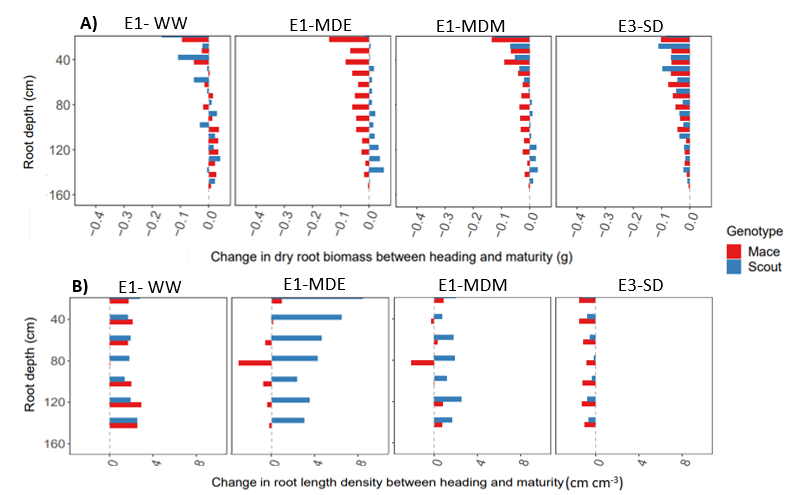


**Supplementary Figure S4.** Change in (A) dry root biomass, (B) root length density and (C) root diameter between heading and maturity at different depths (0 to 150 cm) in Mace and Scout for all studied water treatments. This figure presents data from Figure 8 treatment by treatment.
